# Host-Parasite Interactions in Chagas Disease: Genetically Unidentical Isolates of a Single *Trypanosoma cruzi* Strain Identified *In Vitro via* LSSP-PCR

**DOI:** 10.1371/journal.pone.0137788

**Published:** 2015-09-11

**Authors:** Nívia Carolina Nogueira-Paiva, Paula Melo de Abreu Vieira, Larissa Maris Rezende Oliveri, Kátia da Silva Fonseca, Gwenaelle Pound-Lana, Maykon Tavares de Oliveira, Marta de Lana, Vanja Maria Veloso, Alexandre Barbosa Reis, Washington Luiz Tafuri, Cláudia Martins Carneiro

**Affiliations:** 1 Laboratório de Imunopatologia, Núcleo de Pesquisas em Ciências Biológicas, Instituto de Ciências Exatas e Biológicas, Universidade Federal de Ouro Preto, Ouro Preto, MG, Brazil; 2 Laboratório de Doença de Chagas, Núcleo de Pesquisas em Ciências Biológicas, Instituto de Ciências Exatas e Biológicas, Universidade Federal de Ouro Preto,Ouro Preto, MG, Brazil; 3 Departamento de Ciências Biológicas, Núcleo de Pesquisas em Ciências Biológicas, Instituto de Ciências Exatas e Biológicas, Universidade Federal de Ouro Preto, Ouro Preto, MG, Brazil; 4 Departamento de Farmácia, Escola de Farmácia, Universidade Federal de Ouro Preto, Ouro Preto, MG, Brazil; 5 Departamento de Análises Clínicas, Escola de Farmácia, Universidade Federal de Ouro Preto,Ouro Preto, MG, Brazil; Universidad Nacional Autonoma de Mexico, MEXICO

## Abstract

The present study aims at establishing whether the diversity in pathogenesis within a genetically diverse host population infected with a single polyclonal strain of *Trypanosoma cruzi* is due to selection of specific subpopulations within the strain. For this purpose we infected Swiss mice, a genetically diverse population, with the polyclonal strain of *Trypanosoma cruzi* Berenice-78 and characterized *via* LSSP-PCR the kinetoplast DNA of subpopulations isolated from blood samples collected from the animals at various times after inoculation (3, 6 and 12 months after inoculation). We examined the biological behavior of the isolates in acellular medium and *in vitro* profiles of infectivity in Vero cell medium. We compared the characteristics of the isolates with the inoculating strain and with another strain, Berenice 62, isolated from the same patient 16 years earlier. We found that one of the isolates had intermediate behavior in comparison with Berenice-78 and Berenice-62 and a significantly different genetic profile by LSSP-PCR in comparison with the inoculating strain. We hereby demonstrate that genetically distinct *Trypanosoma cruzi* isolates may be obtained upon experimental murine infection with a single polyclonal *Trypanosoma cruzi* strain.

## Introduction

More than a hundred years after the first human clinical case of Chagas disease was described, the origin of the broad clinical spectrum of the disease, ranging from asymptomatic to cardiac and gastro-enteric manifestations, has yet to be elucidated. The polymorphism of *Trypanosoma cruzi (T*. *cruzi)*, the etiologic agent of Chagas disease, was demonstrated via both biochemical and genetic assays [1]. However, attempts to correlate the pathogenicity, tissue-tropism or drug-susceptibility [2] to specific strains or distinct typing units (DTU) of *T*. *cruzi* have failed.

Polyclonal strains are commonly found in natural infections. They consist of a combination of subpopulations of clones with distinct biological and genetic characteristics. According to the clonal-histotropic model [3] some clonal associations are selectively advantageous resulting in stable strains. The host genetic background may however determine its susceptibility towards infection and towards the development of the disease [4, 5]. When mammals are infected, the clonal repertory may determine which tissues will be affected [6]. Given that not all clones are able to trigger infection, the clones that are able to establish themselves probably compete among each other and colonize distinct tissues. Therefore the interaction between each clone and a specific tissue may be a determining factor in the development of distinct clinical forms of Chagas disease [3].

The Berenice strain was isolated from the first described human case of Chagas disease [7] from a patient living in an endemic area for the disease (município de Pirapora, MG/Brasil). In addition to the *T*. *cruzi* subpopulation isolated during the acute phase [7], another two parasite samples, currently named Berenice 62 (Be-62) and Berenice-78 (Be-78) strains, were isolated from this same patient with a 16-year interval between collections via xenodiagnostic using *Triatoma infestans* and *Dipetalogaster maximus*, respectively [8, 9]. Interestingly both isolates present different *in vitro* biological behavior, virulence and pathogenicity [10]. Two hypotheses have been put forward to explain the alteration in profile of these strains. Even though the patient denies any contact with the vector after contracting the disease, the possibility of re-infection cannot be completely discarded given that she continued living in an endemic area. However, should there have been no re-infection, the differences between the two isolates suggest selection of one or more subpopulations from the initial strain via immunologic mechanisms that take place during the chronic phase of the disease [10]. This hypothesis is supported by the demonstrated polyclonality of the Berenice strain [11] but remains to be elucidated.

While genetic studies can demonstrate the intraspecific heterogeneity of the parasite, probing the biological behavior and host-parasite interactions help clarify the importance of different strains and different subpopulations within a strain in determining the clinical and epidemiologic manifestations of the disease [12]. In this respect, Vago et al. [13] demonstrated that *T*. *cruzi* with distinct kinetoplast DNA (kDNA) signatures were detected in different tissues in human cases of Chagas disease. Veloso and coworkers [14] studied the host-parasite interaction in experimental infections in dogs with polyclonal *T*. *cruzi* strains and found distinct *T*. *cruzi* populations in each host, which composition could vary along the course of long-term infection [14]. Rodrigues and coworkers [5] observed that the immune response of human patients in the case of co-infection with two different *T*. *cruzi* strains does not follow the pattern of the immune response observed in the case of infection with either of the two strains studied. All the above-cited studies of natural and experimental co-infections have pointed out the need to consider clonal interactions in understanding the pathology.

Due to possible adaptive clonal selection, *in vitro* and *in vivo* studies on the genetic variability of *T*. *cruzi* in infected animals have long suffered from experimental limitations related to culture and maintenance of the parasite [15, 16, 17]. Many of these limitations were overcome recently with the introduction of the experimental technique low-stringency single specific primer polymerase chain-reaction (LSSP-PCR), a PCR-based technique that enables DNA profiling directly from an infected tissue [18]. The technique is designed in such a way that minute alterations in the genome, such as a single-base alteration can be detected [19]. As such it is a very sensitive technique capable of differentiating between subpopulations within a given DTU [20].

Although there is supporting evidence for the validity of the clonal-histotropic model, it remains to be confirmed whether a single subpopulation should display tropism for a specific organ or cell component. In an attempt to validate such hypothesis we designed the presently reported study, where the Swiss mice host serves as a “filter” to isolate a clonal subpopulation with distinct genetic characteristics from the infecting strain.

## Animals, Materials and Methods

All procedures and experimental protocols were conducted in accordance with the directives issued by the Brazilian College of Animal Experimentation (COBEA) and approved by the Ethics Committee in Animal Research at the Universidade Federal de Ouro Preto (UFOP), Minas Gerais, Brazil (protocol number 2011/30, the arrive guidelines checklist in S1 File). Prior to the study, the animals were dewormed and vaccinated against several infectious diseases.

### 2.1 *T*. *cruzi* strains

Be-62 [8] and Be-78 [9] strains were maintained by successive passages in mice. Be-62 and Be-78 belong to DTU TcII [21].

### 2.2 Animals and experimental infection

Twenty-four one-month-old Swiss mice from the Center for Animal Science CCA/UFOP, weighed 23.5–24.5g, were inoculated intraperitoneally with 5.0 × 10^3^ bloodstream trypomastigotes of the Be-78 *T*. *cruzi* strain and maintained at the following environmental conditions: 12 h day/night cycle, temperature 22 ± 2°C, food and water *ad libitum* for 12 months after infection (MAI). The infection and curve of parasitemia were confirmed via examination of fresh blood samples according to the methodology of [22]. The anesthetized mice (ketamine 60 mg/kg and xylazine 7.5 mg/kg, [23]) underwent blood sampling for hemoculture at three, six and twelve MAI, which corresponds to the chronic phase of the disease. Under sterile conditions, 200 μl of blood collected from the orbital plexus of each mouse were added to 15 ml sterile tubes (Falcon, Becton Dickinson, USA) containing liver infusion tryptose (LIT) medium supplemented with 10% inactivated fetal bovine serum (LIT-10% FCS, Liver Infusion Tryptose-10% Fetal Bovine Serum) and stored in a refrigerated B.O.D incubator at 28°C ± 1°C for assessment at 30, 60 and 90 days after collection. During the course of the experiment the animals were monitored daily and not show signs of pain or distress. The criteria used for evaluating their health and welfare were their weight, appetite, water consumption, skin and fur condition, in comparison with a non-inoculated control group. At the end of the study, mice were euthanized in a CO_2_ chamber and all efforts were made to minimize suffering.

Positive hemocultures were maintained in exponential growth in LIT medium-10% FCS, for a short period of time to minimize parasite selection, under the same conditions (28°C ± 1°C) [24] to determine the behavior in acellular culture medium (LIT) or cellular culture medium (Vero cells) and also for obtaining a sufficient number of epimastigotes for molecular characterization. Samples of these cultures were subjected to evaluation of *in vitro* growth kinetics of epimastigotes, incubated in Grace (Grace's Insect Medium, Sigma) to induce metacyclogenesis (*in vitro* infection assay) or washed and harvested for LSSP-PCR. For this purpose the cultures were transferred to 50 ml tubes (Falcon, Becton Dickinson, USA) and centrifuged at 3.400*g* for 30 min at 4°C. The supernatant was discarded and the pellet suspended in 10 ml of sterile 10% PBS (Phosphate-Buffered Saline) at pH 7.2. The cells were washed and centrifuged 3 times (3.400*g* for 10 min, 4°C) in 10% PBS. The epimastigotes obtained were stored in a freezer at -70°C.

### 2.3 Growth kinetics in acellular culture medium

*In vitro* growth kinetic curves of the isolates in acellular culture medium LIT-10% SFB were obtained from a standard inoculum of 1 x 10^6^ parasites / ml in a final volume 3 ml. For this purpose the volume used in the inoculum for each culture was determined with a Neubauer chamber, and the cultures were kept in triplicate in a refrigerated B.O.D incubator at 28°C ± 1°C during the evaluation period. Cell count was carried out daily in a Neubauer chamber under 400x magnification over a period of 20 days [25].

### 2.4 Profile of infectivity and *in vitro* development

The *in vitro* profile of infectivity and growth of the isolates of Be- 78 strain were compared to those of the parental Be-78 strain and of Be-62 strain in Vero cell cultures subjected to infection and assessed after 24, 48 and 72h, in triplicate, according to a methodology adapted from Andrade et al. [26]. Briefly, after 24h of culture, semiconfluent monolayers of Vero cells (10^4^) fixed on glass coverslips were incubated at 5% CO_2_ and 37°C for 18h (overnight) with axenic cultures metacyclic trypomastigotes, to a MOI (multiplicity of infection) of 10, of different *T*. *cruzi* subpopulations (10^5^), previously submitted to metacyclogenesis in Grace medium. After washing with PBS to remove extracellular parasites, cultures were kept under the same incubation conditions until the time of collection of the cells (24, 48 and 72h after inoculation). Subsequently, the cultures were fixed in methanol and stained with Fast Panoptic and the ratios of infection and intracellular development or proliferation were examined by optical microscopy under 1000x magnification. The infectivity ratio was calculated as the number of infected cells per 100 cells counted, while the intracellular development ratio was calculated as the ratio of the number of parasites counted over the number of infected cells.

### 2.5 Gene Signature by LSSP-PCR

The evaluation of gene polymorphism between different isolates and the Be-78 parental strain by LSSP-PCR consisted in obtaining, purifying and amplifying the template DNA followed by a second amplification using a single primer under conditions of low stringency [19]. The specific amplification of the 330bp (base pairs) fragment of the kDNA of *T*. *cruzi* was performed according to the method of Gomes et al. [27]. The amplified products were revealed on 1.5% agarose gel (1.0% agarose + 0.5% Low Melting Point agarose / Invitrogen) and stained with ethidium bromide. The 330bp fragment, visualized under UV radiation, was removed from the gel using a plastic pipettor. After heating in a thermostated water bath, these fragments were homogenized, diluted in sterile Milli-Q water at a ratio of 1:10 (v/v) and used in a second amplification step. For the LSSP-PCR reactions the DNA samples were re-amplified using a single specific primer (corresponding to the primer S35), termed S35G* (5’-AAA TAA TGT ACG GGG GAG AT-3’). The reaction mixture reached a final volume of 10 μL containing 6.38 μl of sterile milli-Q water; 2.0 μl of sample buffer; 0.2 μl of each deoxynucleotide (dNTP: dATP, dCTP, dGTP, dTTP—Sigma, St. Louis, MO, USA); 0.1 μl of primer S35G * (450μM) and 0.32 μl of Taq DNA polymerase (5U / μl—Go Taq Promega). To this mixture was added 1 μL of DNA diluted at 1:10 (v/v). The amplification was carried out under the following conditions: initial DNA denaturation step at 94°C for 5 min, annealing at 30°C for 1 min, extension at 72°C for 1 min, followed by 40 amplification cycles consisting of a denaturation step at 94°C for 1 min, annealing at 30°C for 1 min, ending with an extension step at 72°C for 10 min. The products of this second amplification were revealed in duplicate on 8% polyacrylamide gel, stained with silver and analyzed for band sharing.

### 2.6 Statistical analyses

For the evaluation of growth profiles in axenic growth medium the area-under-the-curve (AUC) was considered *via* Kruskal-Wallis one-way analysis of variance for non-parametric data followed by Dunn´s post-hoc test for comparison with the reference Be-78 and Be-62 strains. The *in vitro* infection test with Vero cells were compared using Anova two-way analysis of variance followed by Bonferroni post-hoc test for comparison with the reference Be-78 and Be-62 strains. In all cases, differences were considered statistically significant at *p* values lower than 0.05.

## Results

### 3.1 Hemocultures

Out of the twenty-four mice infected with the Be-78 *T*. *cruzi* strain and subjected to blood collection seven presented positive hemocultures at one sampling time at least (Table 1). Although not all animals were found positive for hemocultures, it was possible to isolate parasites at all three sampling times, over a year of infection.

**Table 1:** Hemocultures. Repartition of the positive hemocultures from blood samples collected from mice infected with Berenice-78 *T*. *cruzi* strains at three, six and twelve months after infection.

Hemoculture	Animal	Identification
3MAI	5	Be-78is5-3mai
	21	Be-78is21-3mai
6MAI	1	Be-78is1-6mai
	2	Be-78is2-6mai
	5	Be-78is5-6mai
	15	Be-78is15-6mai
12MAI	1	Be-78is1-12mai
	15	Be-78is15-12mai

Be-78: Berenice-78; is: isolate; MAI/mai: months after infection.

### 3.2 Growth kinetics in acellular culture medium

**Fig 1:**
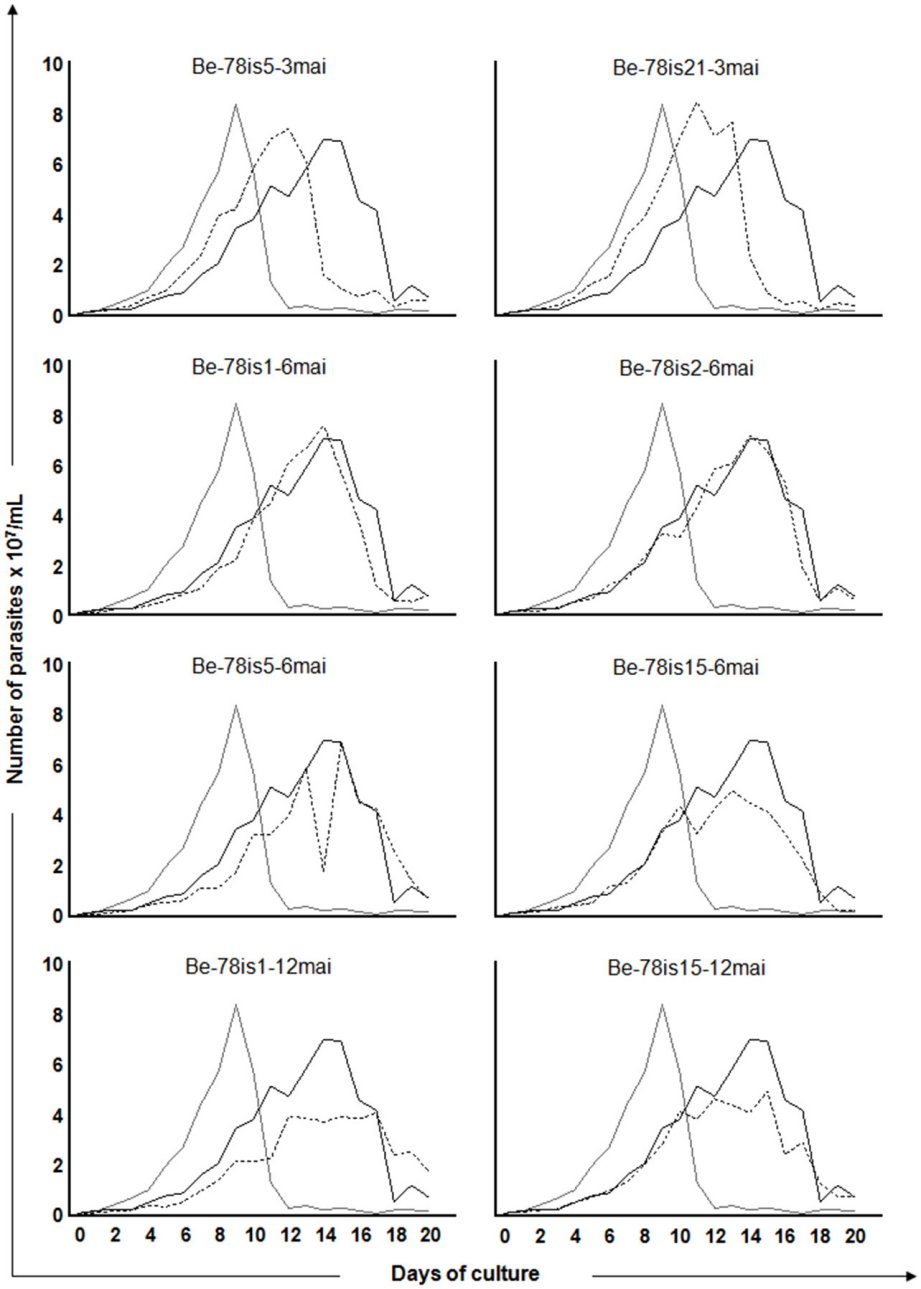
Growth kinetics in acellular culture medium. Growth kinetics of epimastigotes in LIT medium from days 0 to 20 of the parasites isolates obtained from hemoculture at 3, 6 or 12 months after infection of Swiss mice with 5000 blood trypomastigotes from Be-78 *Trypanosoma cruzi* strain. The Y-axis represents the number of parasites triplicate median x 10^7^/ml and the X axis represents the days of culture. The isolates’ growth curves (hatched line) are presented in comparison with the curves of the strain used in the inoculum (Berenice-78 parental: continous black lineBe-78) and of Berenice-62 strain (continous grey line Be-62).

Growth curves were recorded over 20 days after inoculation of 10^6^ parasites / ml of culture for each isolate. We observed a significant difference in the area under the curve (AUC) between the two strains used as reference. The Be-78 parental strain (5,3x10^8^) presented a higher replication ratio than the Be-62 strain (3,7x10^8^). In addition to the AUC, the differences in behavior in the distinct phases of the growth epimastigote curve was confirmed between these strains in axenic culture showing a higher and earlier growth with early entry into the decline phase for the Be-62 strain, compared to the strain Be-78 (Fig 1), as described by other authors [9, 10]. Furthermore, a significant reduction in the AUC was identified in two isolates, Be-78is15-6mai (4,2x10^8^) and Be-78is1-12mai (4,2x10^8^) in comparison with the Be-78 strain used in the inoculum (5,3 x10^8^), indicating that the former have reduced ability to grow in culture medium compared to the parental strain. In comparing the different stages of growth (lag, log, stationary and decline phases), regardless of the AUC, among the various isolates, it was found that both isolates collected at three MAI (Be-78is5-3mai and Be-78is21-3mai) presented profiles similar to the Be-62 strain (Fig 1). This data indicates the presence in the population of strain Be-78 of at least one subpopulation that shares features similar to strain Be-62.

### 3.3 Infectivity and *in vitro* development

**Fig 2:**
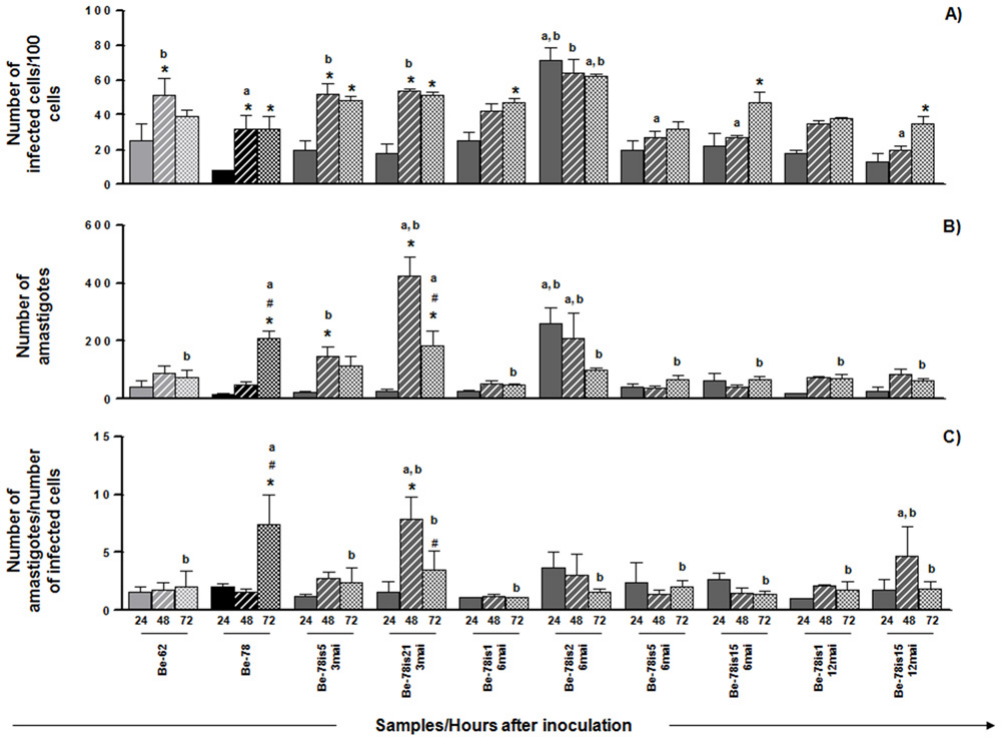
Infectivity and in vitro development. *In vitro* infection assay in Vero cell cultures using Berenice-62 (grey square), Berenice-78 (parental, black square) strains and subpopulations/isolates of Be-78 strain, obtained from hemocultures at 3, 6 or 12 months after infection of Swiss mice with 5000 blood trypomastigotes from Be-78 *Trypanosoma cruzi* strain. After 18 exposure hours to the parasite, the cells were washed to remove extracellular parasites and maintained in medium until collection, fixation and staining 24 (solid bar), 48 (hatched bar) ou 72 (dotted bar) hours after inoculum. A) number of infected cells in 100 counted cells; B) number of intracellular amastigotes in 100 counted cells; C) number of intracellular parasites per infected cell. *: Significant difference compared to the 24 hours of culture; #: Significant difference compared to the 48 hours of culture; a: significant difference compared to the strain Be-62; b: significant difference compared to the Be-78 parental strain. The data represent the mean of triplicates ± standard error.

**Fig 3:**
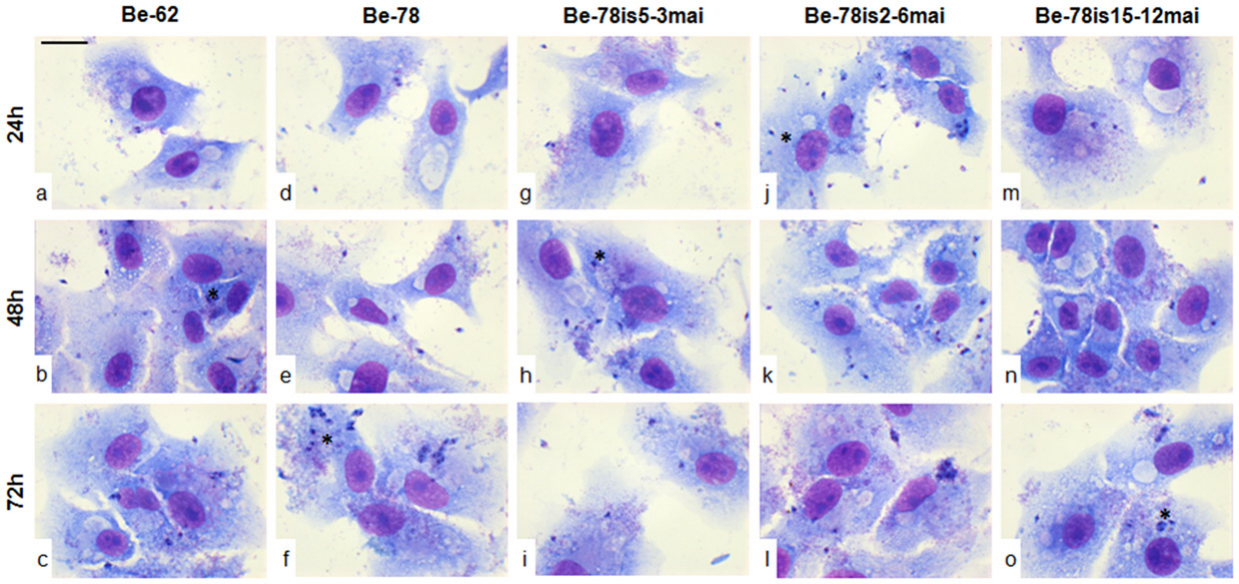
Infectivity and *in vitro* development illustration. Representative photomicrographs of *in vitro* assay infection in Vero cell cultures with Berenice-62 and Berenice-78 parental strains and subpopulations/isolates of Be-78 strain, obtained from hemocultures at 3, 6 or 12 months after infection of Swiss mice with 5000 blood trypomastigotes from Be-78 *Trypanosoma cruzi* strain. After 18 hours exposure to the parasite, the cells were washed to remove extracellular parasites and maintained in medium until collection, fixed and stained at 24, 48 or 72 hours after inoculation. Increasing the rate of infectivity (asterisks) 48h after incubation for Be-62 strain (a, b, c) strain and for Be 78is5-3mai isolate (g, h, i); Increased rate of intracellular multiplication (asterisks) in 72h time for Be-78 parental strain (d, e, f); Infectivity and intracellular development profiles upper (asterisks) for isolate Be-78is2-6mai (j, k. G), and lower for Be-78is14-12mai (m, n, o). Fast Panotic. Bar = 25μm.

In order to evaluate the behavior of each parasite isolate in cell medium, the profiles of infectivity and development in Vero cell were determined at 24, 48 and 72h of incubation (Figs 2 and 3). All subpopulations successfully infected Vero cells after 24h. For most samples the infectivity ratios doubled at 48h after inoculation, corresponding to the period of multiplication of the parasite inside the host cell. One isolate obtained at 6MAI, however, (Be-78is2-6mai) presented ratios of infectivity and development threefold higher than the other isolates within 24h of incubation only, remaining stable until the end of the experiment. Between 48 and 72h after incubation the ratios of infectivity and development of all samples remained stable, except for the ratio of development of the parental strain, lower in the early times, which continued increasing with time, suggesting a slow behavior in cell culture of the latter. All other subpopulations presented an increase in the ratio of infectivity with time and variable ratio of development along the experiment.

Comparison of the *in vitro* ratios of infectivity and intracellular development of the different isolates with the strains used as references in the present study (Be-62 and Be-78) is presented in Fig 2. 48 h after inoculation Be-62 strain and the isolates Be-78is5-3mai and Be-78is21-3mai were found to have parasitized more cells than Be-78 strain.

At 72h after infection, although there was no difference in the infectivity ratios, the ratio of development of amastigotes from Be-78 was higher than that of Be-62 (p<0,001) and of the isolates obtained at three MAI (p<0,05). This data demonstrates that strain Be-62 presents an early increase in infectivity, whereas Be-78 shows a higher ability for *in vitro* development and a slower profile of infectivity. The behaviors of isolates Be-78is5-3mai and Be-78is21-3mai were similar to that of strain Be-62, which is in line with observations from the growth in acellular medium assay.

Isolate Be-78is2-6mai showed a higher infectivity ratio than both strains Be-62 (p<0,001) and Be-78 (p<0,001) already at 24h after inoculation, and remained stable for the rest of the experiment. At 24 and 48h the number of amastigotes was higher than for Be-62 and Be-78. Although the number of intracellular amastigotes per infected cell remained the same between 24 and 48h, the higher number of infected cells for this isolate indicates a fast and efficient infectivity profile. 72 h after inoculation the number of infected cells for isolate Be-78is2-6mai became lower than that of Be-78, which is due to a higher intracellular development ratio of Be-78. Nonetheless, the higher infectivity ratio maintained by isolate Be-78is2-6mai reaffirms the success of this particular isolate in invading new cells of the host tissue. This behavior may be a result of selection by the vertebrate host immune system (Swiss mouse), which in the case of this particular animal promoted a more virulent subpopulation.

The other isolates presented no significant change in infectivity profiles compared to those of Be-62 and Be-78 strains, even though their ratios of development were lower. One exception, however, is the increase in ratio of development, observed 48h after inoculation, for isolate Be-78is15-12mai compared to Be-78 and Be-62, showing an early proliferation peak in comparison with Be-78 and greater replicative capacity compared to Be-62. This isolate showed a growth curve in acellular medium significantly lower than that observed for the Be-78 parental strain, highlighting the variable behavior of each strain under different environmental pressures.

### 3.4 kDNA signature by LSSP-PCR

**Fig 4:**
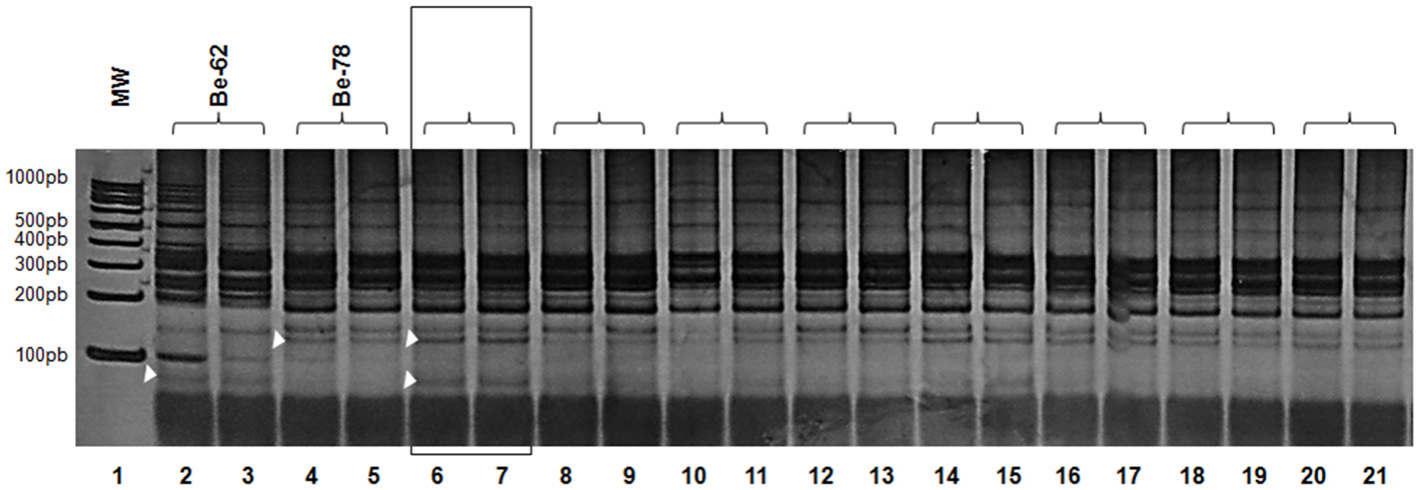
kDNA signature by LSSP-PCR. Gene signatures of 330bp fragment of the kDNA minicircle region revealed in 8% polyacrylamide gel and silver stained. 100bp molecular weight (MW) in channel one and other channels regarding the isolates genetic profiles obtained from hemocultures at 3, 6 or 12 months after infection of Swiss mice with 5000 blood trypomastigotes from Be-78 *Trypanosoma cruzi* strain compared to the Be-78 parental and Be-62 strains. Be-62 (2–3); Be-78 parental (4–5); Be-78is5-3mai (6–7); Be-78is21-3mai (8–9); Be-78is1-6mai (10–11); Be-78is2-6mai (12–13); Be-78is5-6mai (14–15); Be-78is15-6mai (16–17); Be-78is1-12mai (18–19); Be-78is15-12mai (20–21). Arrow heads indicate the bands shared between the isolate Be-78is5-3mai and the reference strains, Be-78 parental and Be-62.

Many of the bands in the multiband patterns by LSSP-PCR of the reference Be-78 and Be-62 strains were composed of bands shared by both reference strains. One band, however, in the 100bp region, is absent from the profile of strain Be-78 and visible in the Be-62 band profile (Fig 4). Isolate Be-78is5-3mai presented a profile with bands shared with each of the strains, the parental strain Be-78 and strain Be-62, including the band slightly below 100bp specific to Be-62. This band was also visible, although with a lesser intensity, in the profiles of two other isolates (Be-78is1-6mai and Be-78is5-6mai), indicating some similarities between the subpopulation(s) present in the isolates and those present in strain Be-62. A slight variability in the band profiles between duplicates may be due to the fact that the isolates were not obtained via cloning and therefore may be composed of more than one subpopulation.

**Fig 5:**
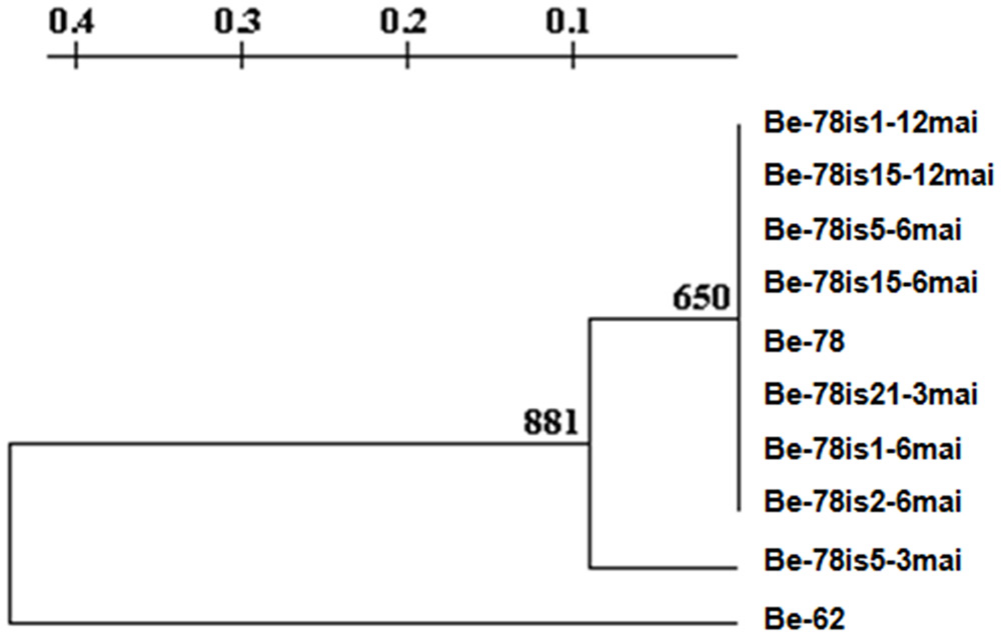
UPGMA. Phenogram constructed from UPGMA (Unweighted Pair Group Method using Arithmetic averages) resulting from the gene signature profile analysis obtained by LSSP-PCR of isolates obtained from hemocultures at 3 (Be-78is5-3mai, Be-78is21-3mai), 6 (Be-78is1-6mai; Be-78is2-6mai; Be-78is5-6mai; 78is15-6mai-Be) and 12 (Be-78is1-12mai; Be-78is15-12mai) months after infection of Swiss mice with 5000 blood trypomastigotes from Be-78 *Trypanosoma cruzi* strain in comparison with the Be-78 parental and Be-62 strains.

Eight bands obtained in the gene signature profile analysis for LSSP-PCR were used to build the phenogram from UPGMA (Unweighted Pair Group Method using Arithmetic Averages). The choice of the bands was based on its resolution, reproducibility between duplicates and intensity. The phenogram showed two distinct groups (Fig 5), group I, including the Be-78 parental strain and the isolates obtained at different times along the experimental chronic phase (Be-78is5-3mai, Be-78is21-3mai, Be-78is1-6mai, Be-78is2-6mai, Be-78is5-6mai, Be-78is15-6mai, Be-Be-78is1-12mai and 78is15-12mai) and group II, comprising the Be-62 strain. In group I, the Be-78 strain of subpopulations divided into two groups, with isolated Be-78is5-3mai, being the one who distanced itself from parental strain. The remaining isolates formed a group more genetically correlated with the parental strain (Fig 5).

Isolate Be-78is5-3mai shared four bands in its LSSP-PCR profile with that of Be-78, versus three with Be-62. Strain Be-78 shared only 2 bands with strain Be-62. This data indicates that the isolate Be-78is5-3mai is genetically closer to strain Be-78 than to strain Be-62, however, this genetic distance between the isolate and Be-62 strain is less than the distance between strain Be-78 and strain Be-62 (Fig 5).

## Discussion

According to the current classification of *T*. *cruzi* subpopulations in Discrete Typing Units (DTU) both Be-62 and Be-78 strains belong to DTU TcII [21]. Although these two strains were sufficiently related to be classified within the same DTU they are morphologically distinct. The Be-62 strain is predominantly composed of slender forms, presents peak parasitemia around the seventh day and a mortality rate of 100% at 15 days after infection in mice [9, 10, 28], while the Be-78 strain is composed of large shapes, presents peak parasitemia later (day 15) and a survival rate of 100% [9, 10, 29, 30]. When introduced in acellular culture medium, Be-78 showed lower growth capacity and high rates of differentiation, while under the same conditions Be-62 showed a high capacity of multiplication and average rates of differentiation [9, 10].

Furthermore, data obtained in our laboratory showed that strains belonging to the same DTU, namely TcII, Y and Be-78 strains, had drastically different pathogenicities in experimental Beagle dog infection. Animals infected with the Y strain showed controlled parasitism and their immune system returned to homeostasis, whereas animals infected with the Be-78 strain maintained parasitism over two years after infection and showed lesions of apparently progressive character [31]. The interaction of each subpopulation within the strain with the host may interfere with the development of the host immune response and determine success in controlling tissue parasitism and associated lesions.

In order to test the ability of an individual to select a clonal subpopulation from a polyclonal strain of *T*. *cruzi* we infected 24 Swiss mice with strain Be-78 and tested blood samples at three different times after infection. The parasites from positive hemocultures were characterized *in vitro* in terms of growth kinetics in acellular medium, profile of infection and development in Vero cell medium and kDNA gene-signature by LSSP-PCR. Swiss mice were selected for the present study, rather than a clonal population of mice, because of their genetic variability. It was already observed that upon infection with a polyclonal strain of *T*. *cruzi* the host-parasite interaction may lead to the absence of parasitemia in some individuals, whereas individuals with positive hemocultures may differ in terms of pathologic response, in particular tissue-tropism [4], most likely due to genetic factors.

We found that the gene signatures of Be-62 and Be-78 obtained by LSSP-PCR were different, confirming the genetic heterogeneity of the two strains isolated from the same patient with a 16 years interval. Our data complements previous literature reports using isoenzyme profiles, RAPD and microsatellites [9, 10, 11, 32]. The Be-62 and Be-78 strain samples have been maintained in our laboratory for the past 30 years by alternating passages in mice and axenic culture, and including periods of cryopreservation. It is worth mentioning that, in spite of alternating between *in vivo* and *in vitro* maintenance, no changes in the virulence, pathogenicity or molecular profile have been observed in comparison with the original samples, as demonstrated in various studies published by our group [9, 11, 14, 17, 29; 33; 34].

The kDNA profile of one of the isolates, Be-78is5-3mai, was closer to Be-62 and Be-78 strains (Fig 3). Interestingly, isolate Be-78is5-3mai, showed a behavior in Vero cell culture medium closer to that of the Be-62 strain. In contrast, isolate Be-78is2-6mai, which showed *in vitro* ratios of infectivity and development threefold higher than the Be-78 parental strain was genetically similar in terms of gene signature by LSSP-PCR, showing that changes in the biological behavior do not necessarily reflect genetic variations. Growth in culture medium simulates the behavior of epimastigotes in the gut of an invertebrate host and, therefore, a low ratio of infectivity or development may reflect a reduced ability for multiplication of these subpopulations, with potential implications on the success of vector infection.

The isolate that stood out the most in terms of genetic distance to the parental strain, Be-78is5-3mai, was collected from animal 1 at 3MAI. Another isolate was obtained from the same animal three months later at 6MAI, Be-78is5-6mai. Although the latter also displayed in its LSSP-PCR profile the characteristic band slightly below 100bp similar to Be-62, yet with a lower intensity, its biological properties *in vitro* were similar to the parental strain Be-78. This observation is a hint on the reversibility of the genetic modulation over time in the case of chronic infection with *T*. *cruzi*, where a subpopulation may be favored at a certain time and another one take over later on.

Even though maintenance of the isolates in culture medium was limited in time, it is important to consider the possible influence of hemoculture and *in vitro* maintenance prior to phenotypic and molecular characterization, which could add up to clonal selection exerted by the immune system of the vertebrate host (Swiss mice). Hence, hemoculture and *in vitro* maintenance may favor one or more subpopulation over others resulting in the expansion or reduction of selected clones that were present in the original sample [35]. However, this process not allow the appearance of subclones that were absent from the original sample. Therefore it is more likely that the selective process, which resulted in isolate Be-78is5-3mai was a consequence of the pressure exerted by the immune system of the host rather than a consequence of clonal selection during *in vitro* maintenance.

Microsatellite characterization of the Be-78 strain by Valadares et al. [11] confirmed its multiclonality, and suggested the presence of a subpopulation with similar characteristics to those of strain Be-62. Therefore, isolate Be-78is5-3mai may have originated either from selection of a subpopulation within the polyclonal parental strain Be-78, or due to the association of subclones, with the participation of one or more subclones, resulting in mixed biological and molecular profiles. The latter is supported by the isolate´s intermediate behavior in culture medium and genetic profile in comparison with the reference strains, Be-78 and Be-62.

The present data confirms the polyclonal or mixed nature of strain Be-78 already reported in the literature [11, 14, 17] and reinforces the hypothesis that the selective pressure exerted by the host throughout the chronic phase may favor the development of one or more subpopulation(s) over the other(s). This phenomenon may be related to the plasticity of *T*. *cruzi* and not less importantly, to the parasite-host interaction, since all outbred animals received the same inoculum and responded to infection in selecting distinct subpopulations.

In conclusion, three isolates were obtained upon infection of Swiss mice with Be-78, namely Be-78is5-3mai, Be-78is2-6mai and Be-78is15-12mai, which showed differences in biological and/or molecular parameters compared to the parental strain. As such, they are good candidates for probing *in vivo* the influence of clonal subpopulations in the pathogenesis of the disease.

## Conclusion

*T*. *cruzi* isolates from Swiss mice infected with the same polyclonal parental strain Be-78 characterized *in vitro* were found to have a kDNA gene signature significantly distinct from the parental strain. The present work confirms the hypothesis that the maintenance of a *T*. *cruzi* population in outbred mice results in the selection of genetically distinct subpopulations.

Subsequent *in vivo* studies are under way to evaluate the pathogenicity and histotropy of a specific isolate in comparison with that of the parental strain in experimental murine infection.

## Supporting Information

S1 File The ARRIVE Guidelines Checklist. **R**eport of *in vivo* experiments in animal research.
(PDF)
